# Variation between nursing homes in drug use and in drug-related problems

**DOI:** 10.1186/s12877-020-01745-y

**Published:** 2020-09-09

**Authors:** Amura Francesca Fog, Ibrahimu Mdala, Knut Engedal, Jørund Straand

**Affiliations:** 1 Nursing Home Agency, Oslo Municipality, Norway; 2 General Practice Research Unit, Department of General Practice, Institute of Health and Society, University of Oslo, Postbox 1130 Blinderen, N-0318 Oslo, Norway; 3 Norwegian National Advisory Unit for Aging and Health, Vestfold County Hospital HF, Toensberg and Oslo University Hospital, Oslo, Norway

**Keywords:** Older people, Nursing homes, Medication review, Psychotropic drugs, Opioids, Drug related problems

## Abstract

**Background:**

Residents at nursing homes (NHs) are at particular risk for drug related harm. Regular medication reviews using explicit criteria for pharmacological inappropriateness and classification of drug related problems (DRPs) have recently been introduced as measures to improve the quality of medication use and for making the treatment more uniform across different institutions. Knowledge about variation in DRPs between NHs is scarce. To explore if increased attention towards more appropriate drug treatment in NHs have led to more uniform treatment, we have analyzed variations between different nursing homes’ drug use and DRPs.

**Methods:**

Cross-sectional medication review study including 2465 long-term care residents at 41 NHs in Oslo, Norway. Regular drug use was retrieved from the patients’ medical records. DRPs were identified by using STOPP/START and NORGEP criteria and a drug-drug interactions database. NHs were grouped in quartiles based on average levels of drug use. The upper and lower quartiles were compared using independent samples t-test and associations between drug use and DRPs were tested by logistic regression.

**Results:**

Patients’ mean age was 85.9 years, 74.2% were women.

Mean numbers of regular drugs per patient was 6.8 and varied between NHs from 4.8 to 9.3.

The proportion of patients within each NH using psychotropic and analgesic drugs varied largely: antipsychotics from three to 50%, benzodiazepines from 24 to 99%, antidepressants from nine to 75%, anti-dementia drugs from no use to 42%, opioids from no use to 65% and paracetamol from 16 to 74%.

Mean DRPs per patient was 2.0 and varied between NHs from 0.5 to 3.4.

The quartiles of NHs with highest and lowest mean drugs per patient (7.7 vs. 5.7, *p* < 0.001) had comparable mean number of DRPs per patient (2.2 vs. 1.8, *p* = 0.2). Using more drugs and the use of opioids, antipsychotics, benzodiazepines and antidepressants were associated with more DRPs.

**Conclusions:**

The use of psychotropic and analgesic drugs was high and varied substantially between different NHs. Even if the use of more drugs, opioids and psychotropic drugs was associated with DRPs, no difference was found in DRPs between the NHs with highest vs. lowest drug use.

## Background

Residents in nursing homes (NHs) are often old and due to multimorbidity and frailty have short life expectancies and extensive needs for assistance for carrying out activities of daily living. Dementia and BPSD (Behavioural and Psychological Symptoms in Dementia) represent the most significant mental health challenges in the NH setting affecting respectively 80 and 72% of the residents [1]. Due to multiple diagnoses and symptoms, NH residents often use many drugs and in Norway during the last decades, the use of regular drugs has increased from about five to eight drugs per NH resident [2, 3]. The use of psychotropic drugs [4] and opioids [5] has increased, except for the prevalent use of antipsychotics that now seem to decline [6]. About one in five residents, uses more than one psychotropic drug at the same time [6], in most cases as long-term treatment for BPSD [7].

Due to age-related changes in pharmacokinetics and pharmacodynamics, frail and old people are at higher risk for drug related harms [8] and the presence of dementia ads further to this risk due to impaired ability to communicate drug effects. The widespread use of antipsychotic drugs, benzodiazepines and antidepressants for BPSD is largely inappropriate, because they are commonly used instead of recommended non-pharmacological interventions [9, 10], they have limited effects and their use is associated with an increased risk for adverse drug reactions like delirium, impaired balance and falls and stroke [11]. Substantial variations in drug use have previously been reported among residents in otherwise similar NHs with comparable patient populations [12–15], even if located in the same geographical area [13], and that institutions with high prevalence of drug use tend to use higher dosages [14], probably due to different prescription cultures and organizational factors at the institutions.

Potentially inappropriate medications (PIM), as defined by explicit criteria [16] are common in NHs [17]. In Norway, medication reviews (MRs) are now recommended for the identification of drug related problems (DRPs) among NH residents [18]. The Norwegian national guidelines on dementia also recommend that in NHs, MRs should be done at least once every year [9]. DRP, defined as “an event or circumstance involving drug therapy that actually or potentially interferes with desired health outcome” [19], are identified by using explicit criteria for pharmacological inappropriateness and drug-drug interaction databases.

According to previous studies, DRPs are common in the NH-setting [2, 3, 13]. However, little is known about the variation in DRPs between comparable NHs and how this variation relates to corresponding variations in drug use [20].

Based on a cross sectional study in 41 NHs with 2465 residents [21], we aim to describe the variation between the NHs with respect to their drug use (in particular for psychotropic drugs and analgesics) and corresponding variation in DRPs, and to explore the associations between the two.

## Methods

This is a clustered (by NH) cross-sectional study of the baseline data from a multidisciplinary MR project in 41 NHs (2465 long-term care patients) in Oslo, Norway, that took place during November 2011 and February 2014 [21].

The NHs were recruited by invitation. Of the 51 NHs in Oslo municipality with long-term patients (*n* = 4020), 41 NHs accepted to performed MRs at one, several or all the bed units in their institutions. All patients, and next of kin for patients with dementia at the participating bed units, were asked to participate in the study (*n* = 2625 patients) with the exception of those terminally ill. Eighteen patients refused and 142 scheduled MRs were not performed because the patient died (*n* = 32), became terminally ill (*n* = 33), moved to another NH (*n* = 18) or due to logistical reasons (*n* = 59) during the study period. In average 60 patients per NH (range 19–136 patients per NH) underwent MR. The MRs were conducted as a structured evaluation of each patient’s entire drug use and the assessment of DRPs was standardized across the NHs. Training sessions were held for the involved physicians, nurses and pharmacists (*n* = 5) before project start.

At each NH, a multidisciplinary panel made up by the responsible physician and nurse from the NH together with an externally hired clinical pharmacist, performed MRs according to a standardized procedure in line with the national guideline for MRs [18]. Medication lists for about eight patients were reviewed at each meeting that lasted about 2 h. Prior to the MR meetings, and based on anonymized medication list, the pharmacist collected data on the drugs used and reviewed the medication charts to identify possible DRPs by using the explicit criteria for pharmacological inappropriateness STOPP/START [22] and the Norwegian NORGEP criteria targeting population 70 years and older seen in primary care [23], as well as the national drug-drug interaction database [24]. At the review meetings, the panel assessed the drug use and possible DRPs taken into consideration clinical information (e.g., diagnoses, lab-tests) from the patient’s medical record. The panel then agreed upon and classified the DRPs according to a national consensus classification system [25]. Six DRP categories were applied: 1) Drug choice problem (with subcategories 1a) need for additional drug, 1b) unnecessary drug, 1c) inappropriate drug choice); 2) Dosing problem (with subcategories 2a) too high, 2b) too low, 2c) sub-optimal dosing scheme, 2d) sub-optimal formulation); 3) Adverse drug reactions; 4) Interactions; 5) Inappropriate drug use (with subcategories 5a) administered by health personnel, 5b) administered by patient) and 6) Other (with subcategories 6a) monitoring required, 6b) unclear documentation, 6c) not classified). In case of disagreement, the physician held the final decision.

For each patient we retrieved the following variables from the baseline-data of the MR project: patient’s age, gender, regularly used drugs (name, ATC-code [26], DRPs (category and drug involved), NH identification number, residency at regular (RU) or special care unit for dementia (SCU), and the pharmacist involved in the MR. We especially focussed on the use of psychotropic and analgesic drugs because their use, although largely considered potentially inappropriate [9, 10], has increased in NHs [4, 5] and because they are frequently involved in DRPs [3, 13, 14, 20, 21]. Psychotropic drugs comprise antipsychotics (ATC code: N05A), benzodiazepines (anxiolytics N05B and hypnotics/sedative N05C), antidepressants (N06A), and antidementia drugs (N06D). Analgesics comprise opioids (N02A) and paracetamol (N02B). For each NH we recorded the total number of beds for long-term care and the bed unit mix (RU, SCU or both). All NHs were publicly funded and had comparable staffing of physicians and qualified nurses in line with the county standard; all NHs were non-academic and did not have in-house pharmacists.

### Statistical analyses

Depending on data distribution, numerical data were summarized using mean with standard deviation (SD) or median and range.

For each NH, we calculated the mean number of regular drugs per patient, the mean number of DRPs per patient, the proportion of patients using the targeted psychotropic and analgesic drugs and the proportion of patients exposed to any DRPs. We grouped the NHs into four quartiles, based on their mean number of drugs per patient, the upper quartile comprising those with highest numbers. When a NH was allocated in a particular quartile, data from all residents in that institution were allocated to the quartile. The NHs with highest levels (comprising the upper quartile) were compared to the NHs in the lowest quartile, and mean differences with 95% confidence intervals (CI) were calculated using independent t-test. Relationships between the drug use and the DRPs at the respective NHs were identified using Pearson’s correlation coefficient (r). Counts of DRPs per patient were analyzed using a Poisson regression model with random effects clustered by NH and adjusted for gender and age. We obtained estimates of incidence rate ratios (IRR) from the Poisson regression model, which showed the relative change in counts in one category of a variable relative to the referent category*.* The analyses were performed using Stata SE 15 (Stata Corp LP, College Station, TX) and IBM SPSS Statistics v.24 (IBM Corp., Armonk, NY).

## Results

The 41 NHs had in average 102 beds (range 32 to 185). Seven NHs had only RUs, three NHs had only SCUs and 31 NHs had both types of bed units.

Of the 2465 patients with MR, 1868 were residents living in RUs and 597 at SCUs. The mean age of the residents was 85.9 years (range 36–108 years). The age distribution was comparable across the NHs, except for two institutions especially designed for younger people with dementia (61.3 and 68.4 years, respectively). There were more women (74.2%), who on average were older than men (86.9 vs. 82.8 years). The gender distribution was comparable across the NHs. In total 16,634 drugs were used on a regular schedule, the mean proportion of drugs per patient was 6.8 ± 0.9 and the mean number of drugs per patient varied between the NHs from 4.8 to 9.3. Overall, the most commonly used drugs were for the ‘nervous system’ (2.2 drugs per patient, range of 1.4–3.1) and of these, 2.0 drugs per patient (range 1.3–2.7) were psychotropic and analgesic drugs. At the MR meetings, 4847 DRPs in 84.1% of the patients were identified. Psychotropic drugs and analgesics were involved in 33.9% of all DRPs (Table 1). The most frequent problems were use of unnecessary drug (31.9%), excess dosing (14.2%) and requirement to monitor the drug use (14.2%).

**Table 1:** The drug groups commonly involved in drug-related problems in the total cohort (2465 patients at 41 nursing homes)

Drug-related problems (DRPs)		Drugs		The drug groups commonly involved in the drug-related problems listed			
Categories of DRPs	*n* (%)	ATC-N drugs^a^ *n* (%)	All other drugs *n* (%)	No. 1	*n* of drugs	No. 2	*n* of drugs
Need for additional drug (1a)	372 (7.7)	50 (13.4)	322 (86.6)	B vitamins^b^	155	Iron supplements	39
Unnecessary drug (1b)	1544 (31.9)	474 (30.7)	1070 (69.3)	Benzodiazepines^c^	185	Antidepressants	121
Inappropriate drug choice (1c)	382 (7.9)	131 (34.3)	251 (65.7)	Benzodiazepines	60	Opioids^d^	31
Excess dosing (2a)	688 (14.2)	291 (42.3)	397 (57.7)	Benzodiazepines	110	Proton pump inhibitors	103
Under-dosing (2b)	160 (3.3)	71 (44.4)	89 (55.6)	Opioids	23	Thyroid therapy	23
Adverse drug reaction (3)	276 (5.7)	134 (48.6)	142 (51.4)	Benzodiazepines	63	Antipsychotics	28
Drug–drug interactions^e^ (4)	419 (8.6)	207 (49.4)	212 (50.6)	Antidepressants	115	Antithrombotic agents^f^	55
Monitoring of drug use required (6a)	687 (14.2)	329 (47.9)	358 (52.1)	Antidepressants	105	Antipsychotics	50
Other^g^	364 (6.5)	88 (24.2)	276 (75.8)	Beta-blockers	33	Paracetamol	25
DRPs (total)	4847 (100)	1775 (36.6%)	3072 (63.4%)	Benzodiazepines	489	Antidepressants	456

^a^Psychotropic drugs and analgesics *n* = 1642 (92.5% of all ATC-N drugs)

^b^B12 vitamin, folate and B-complex vitamins

^c^Benzodiazepines comprising anxiolytics (N05B) and hypnotics/sedatives (N05C)

^d^Weak opioids (codeine, tramadol) and strong opioids (N02A)

^e^One drug–drug interaction was recorded as two problems

^f^Mainly warfarin, acetylsalicylic acid and heparin (ATC-B01A)

^g^The remaining DRP categories

**Fig. 1:**
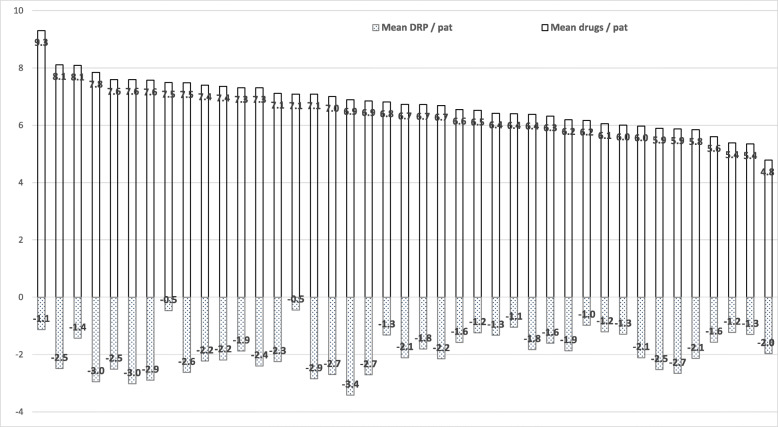
Variation in the number of drugs and of drug-related problems per patient at the 41 nursing homes . Each bar represents one NH with their respective mean drugs per patient (above) and mean DRPs per patient (below the zero line, respectively). The NHs are listed in the same order as in Table 2

The mean number of drugs per patient and the mean number of DRPs per patient at each of the 41 NHs are presented in Fig. 1. (Fig. 1).

The proportion of patients within each NH using different psychotropic drugs varied substantially between the NHs: antipsychotics from 3.0 to 50.0%, benzodiazepines from 23.7 to 98.6%, antidepressants from 9.1 to 75.0%, and antidementia drugs from none to 41.7%. For opioids and paracetamol, the variation in use ranged from respectively no use to 65.2% and from 15.8 to 73.9%. (Table 2) NHs using more drugs also used more opioids (Pearson correlation coefficient *r* = 0.682) and benzodiazepines (*r* = 0.411). Regardless of the total drug use, associations were found between the use of antidepressants and antidementia drugs (*r* = 0.451), opioids and benzodiazepines (*r* = 0.434), opioids and paracetamol (*r* = 0.358), opioids and antidementia drugs (*r* = − 0.315) and between antidementia drugs and antipsychotics (*r* = 0.432).

**Table 2:** Variation in the proportion of patients using psychotropic and analgesic drugs at the 41 nursing homes

Nursing Home	Patients *n*	Antipsychotics *%*	Benzodiazepines *%*	Antidepressants *%*	Antidementia *%*	Opioids *%*	Paracetamol *%*
1	30	23	67	47	7	53	37
2	57	39	98	42	11	51	32
3	23	9	65	57	0	65	74
4	19	37	37	16	5	26	16
5	61	13	39	38	8	30	38
6	49	12	33	53	18	35	73
7	92	21	63	29	13	46	45
8	88	14	78	45	9	61	45
9	88	13	48	30	10	31	51
10	60	30	67	35	15	38	40
11	64	13	64	36	6	50	73
12	72	19	49	50	22	33	49
13	85	18	56	51	7	49	55
14	73	21	63	27	7	51	27
15	94	10	83	28	3	48	66
16	34	12	59	26	9	26	44
17	80	43	84	34	15	31	46
18	136	21	52	39	18	32	44
19	55	4	51	9	0	36	35
20	68	15	59	47	10	26	44
21	66	14	41	29	21	27	41
22	72	18	42	42	14	47	44
23	59	3	53	42	14	39	47
24	38	11	61	32	5	32	45
25	42	31	36	50	12	26	33
26	48	23	29	71	42	19	46
27	78	17	77	36	12	28	44
28	63	13	41	24	10	29	62
29	41	17	46	59	12	27	54
30	52	33	40	50	0	15	54
31	65	11	26	25	14	29	40
32	38	21	24	45	11	37	26
33	24	50	71	75	33	0	21
34	38	21	24	26	3	21	32
35	19	5	53	37	11	21	42
36	53	42	72	28	36	23	34
37	73	12	55	25	8	36	32
38	81	10	42	25	7	23	43
39	61	21	43	28	8	30	34
40	63	16	38	41	2	16	32
41	63	11	37	24	2	14	44

Between the NHs, the mean DRPs per patient varied substantially, from 0.5 to 3.5. The use of unnecessary drugs was associated with excessive dosing (Pearson correlation coefficient *r* = 0.801), inappropriate drug choice (*r* = 0.490) and need for additional drug (*r* = 0.399) at the respective NHs.

The NHs with highest levels of mean drugs per patient (10 NHs, comprising the upper quartile) used more opioid drugs than the NHs with lowest levels of mean drugs/patient (10 NHs, comprising the lower quartile), whereas there were no significant differences in the prevalence of DRPs, except for drug-to-drug interactions. (Table 3).

**Table 3:** Variation between the 41 nursing homes in drug use and drug-related problems and the differences between the quartile of nursing homes using highest and lowest number of drugs

Variables	All NHs (***n*** = 41) Mean (range)	Differences between the NHs using highest (***n*** = 10) and lowest (***n*** = 10) number of drugs			
		Mean Q_4_	Mean Q_1_	Diff (95%CI)^a^	*P-*value
**Drug use**					
Drugs/patient	6.8 (4.8–9.3)	7.7	5.7	2.0 (1.6, 2.6)	< 0.001
Proportion of patients using:					
≥ 9 drugs	34.2 (15.9–52.2)	44.2	22.0	22.2 (18.5, 25.9)	< 0.001
Opioids	33.1 (0.0–65.2)	42.1	22.0	20.1 (8.7, 31.4)	0.002
Paracetamol	43.5 (15.8–73.9)	44.7	34.0	10.6 (−1.5, 22.7)	0.08
Antipsychotics	19.1 (3.0–50.0)	20.3	20.9	- 0.6 (−12.3, 11.0)	0.9
Benzodiazepines	52.8 (23.7–98.6)	57.8	45.7	12.1 (−4.9, 29.1)	0.2
Antidepressants	37.8 (9.1–75.0)	38.2	35.3	2.8 (−10.2, 15.9)	0.6
Antidementia drugs	11.4 (0.0–41.7)	10.7	12.0	- 1.3 (−10.6, 8.1)	0.8
**Drug-related problems (DRPs)**					
Proportion of patients with DRPs	84.1 (31.8–100.0)	85.2	86.2	- 1.0 (−14.9, 12.8)	1.0
DRPs/patient	2.0 (0.5–3.4)	2.2	1.8	0.4 (−0.3, 1.0)	0.2
Categories of DRPs:					
- Unnecessary drug	0.6 (0.1–1.3)	0.6	0.6	0.0 (−0.2, 0.3)	1.0
- Excessive dosage	0.3 (0.1–0.6)	0.3	0.3	0.0 (−0.1, 0.2)	0.2
- Monitor use required	0.3 (0.0–0.7)	0.3	0.3	0.0 (−0.1, 0.2)	0.9
- Need for new drug	0.2 (0.0–0.4)	0.2	0.1	0.1 (−0.01, 0.1)	0.1
- Drug-drug interaction	0.2 (0.0–0.5)	0.2	0.1	0.1 (0.01, 0.2)	0.03
- Adverse drug reaction	0.1 (0.0–0.4)	0.2	0.1	0.1 (− 0.04, 0.2)	0.2
- Inappropriate drug	0.1 (0.1–0.5)	0.1	0.2	- 0.1 (−0.1, 0.1)	0.9
**Demographics**					
Mean age, years	85.9 (61.3–90.0)	84.6	84.5	0.1 (−6.4, 6.7)	1.0
Proportion of males	25.8 (13.6–47.4)	27.5	24.5	3.0 (−6.0, 12.0)	0.5

^a^The mean of the lower quartile (Q_4_) was compared to the mean of the lower quartile (Q_1_) using the Independent samples T test, with difference in means with 95% CI and *p*-value

In the total cohort clustered by NH, using more drugs or being a woman were associated with a 7% [IRR 95% CI: 1.07 (1.06, 1.08), *p* < 0.001] and a 9% [IRR: 1.09 (1.0, 1.2), *p* = 0.007] increase in DRPs, respectively. The use of opioids [IRR: 1.07 (1.0, 1.1) *p* = 0.01], antipsychotics [IRR: 1.20 (1.1, 1.3) *p* < 0.001], benzodiazepines [IRR: 1.08 (1.0, 1.1) *p* = 0.007] and antidepressants [IRR: 1.18 (1.1, 1.2) *p* < 0.001] were associated with an increased risk for DRPs at the respective NHs. Residing at SCU was associated with less DRPs [IRR: 0.85 (0.8, 0.9) *p* < 0.001], whereas age, size of NH or the participating pharmacist (out of in total five) involved in the MRs were not associated with the frequency of DRPs at the NHs.

## Discussion

We found considerable variation in the drug use among the NHs, in terms of number of drugs used on regular basis. This was in particular pronounced for the use of analgesics and psychotropic drugs where the variation was extremely large. We believe that this variation reflect local therapeutic subcultures involving inappropriate drug use. Our findings here represent an important challenge for future quality improvement measures, especially because the psychotropic drugs include risk for many and serious side effects in frail old people with dementia [11]. However, our results are generally consistent with those reported elsewhere for long-term care home residents in Norway [12, 14], Europe [27], US [15, 28] and Canada [29].

Further, the study documented that the rates of DRPs varied up to seven-fold (from 0.5 to 3.4) between the NHs. To the best of our knowledge, only two medication review studies have previously reported variation in DPRs between NHs: one in two urban NHs, from 3.0 to 5.5 mean DRPs per patient [20] and another study in four rural NHs, from 2.7 to 5.6 mean DRPs per patient [30]. The mean of 2.0 DRPs per patient found in the total cohort is below those previously reported in Norway [2, 3, 13], probably because we reported DRPs agreed upon by the team, not all DRPs suggested by the pharmacist.

The associations between the uses of opioids, antipsychotics, benzodiazepines or antidepressants and increased risk of DRPs are consistent with the fact that so many of these drugs are commonly considered potentially inappropriate and should therefore be avoided whenever possible in frail olds. In our study, psychotropic and analgesic drugs were involved in just one third of the total DRPs, and it would be expected that by including also drugs for pro re nata use (“as needed”), this would probably have increased even more the contribution of psychotropic and analgesic drugs to the numbers of DRPs [21]. The correlation between the use of many drugs and more opioids and benzodiazepines at the respective NHs might reflect local prescription cultures [28], or simply a way to relieve staff pressure [31], as prescription of psychotropic drugs and painkillers in combination is not recommended to treat neither pain nor BPSD [9, 32].

We found no difference in the levels of DRPs between the NHs with highest and lowest drug use, although using more drugs was associated with DRPs. This unexpected finding might be due to our analytic strategy by grouping the NHs into quartiles, in addition to a large variation in the levels of DRPs within each group (e.g., three high-drug use NHs with low levels of DRPs and four low-drug use NHs with high levels of DRPs). The strong correlations found between need for additional drug, use of unnecessary drug, excessive dosing and inappropriate drug choice, suggest that prescription quality is multifaceted and hence, in case it is suboptimal, e.g. due to a high rate of DRPs, this will affect several areas of drug prescription practice.

The large difference in DRP levels found between otherwise comparable NHs most probably reflect different institutional prescription cultures, with higher prescription rates at NH-level irrespective of the patient’s clinical indications [29] or different organizational initiatives for patient safety at the NH [33]. To improve the quality of drug use in the NH setting, staff should be educated in geriatric pharmacotherapy and on alternative non-pharmacological interventions [9, 10]. Other measures should include implementing educational programs on person-centred care [34] and multidisciplinary medication reviews [18], which may also include collaboration with a geriatrician [35].

### Strengths and limitations

The strength of this close to practice study was the standardized procedure for MRs, with face-to-face meetings between pharmacist, physician and nurse, having access to patients’ clinical information, and agreeing on actual DRPs for each patient.

It is an important limitation that we have only recorded the DRPs that were accepted by the physicians, without recording all the DRPs that were initially suggested by the pharmacists. Hence, we do not know how the physicians’ acceptance rates varied between the different NHs and how appropriate their rejections were [30]. Some doctors may have experienced suggestions to change their treatment as a threat and criticism towards their own prescribing practice.

The explicit criteria used in this study were updated [16] and tailored for the NH-setting [36] after the study had started, however, we do not believe that using the updated criteria would have changed our results significantly. Instead, it may be questioned if the explicit criteria used were sensitive enough to detect over- and underprescription, or inappropriate medication among multimorbid, frail NH residents commonly exposed to extensive off-label pharmacological treatment for BPSD. Although DRPs, as identified in our study, might have limitations as quality indicators for drug prescription, the NHs with high levels of DRPs probably have proportionally larger potentials for quality improvement.

We believe that the sample of institutions and residents is representative for the long-term care NH-setting because the vast majority of the NHs in the municipality participated in the study. This is a cross-sectional study, and thus we are not able to draw conclusions about causal relationships for the variation. The NHs in Oslo are quite similar: They are publicly financed and administered by the same agency, are non-academic institutions operating in the same regulatory and clinical practice context. They are staffed with full-time nursing home physicians and registered nurses according to the country standard. None of them had an in-house pharmacist. The patient-mix is quite similar due to equal admission criteria. Grouping the NHs in quartiles might be challenged due to the somewhat limited number of NHs.

## Conclusions

Drug use and DRPs varied substantially between comparable NHs. The use of psychotropic and analgesic drugs was high and the unacceptable variation between NHs suggests different and inappropriate drug prescription cultures at several institutions. The use of unnecessary drugs and excessive dosing were common, suggesting overtreatment. There was no difference in DRPs between the group of NHs with highest and lowest drug use, although using more drugs, opioids and psychotropic drugs was associated with an increased risk for DRPs at the respective NHs. Future research on variation between NHs in drug use and DRPs should include variables that describe patient-level factors, such as degree of functional and cognitive impairment of the residents and organizational characteristics, such as leadership, staff number per resident, proportion of registered nurses and postgraduate training of the NH physicians in geriatric pharmacotherapy.

## Data Availability

All data generated or analysed during this study are included in this published article. The datasets used and/or analysed during the current study are available form the corresponding author on reasonable request.

## Abbreviations

ATC: Anatomical Therapeutic Chemical classification system
BPSD: Behavioural and psychiatric symptoms of dementia
DRP: Drug-related problems
IRR: Incidence rate ratios with 95% confidence interval
MR: Medication review
NH: Nursing home
r: Pearson’s correlation coefficient
RU: Regular unit
SCU: Special care unit for dementia
